# Seasonal analysis and environmental risk assessment of selected emerging pollutants in the Vaal River catchment area of South Africa

**DOI:** 10.1007/s10661-025-14649-4

**Published:** 2025-10-29

**Authors:** Zamazwi Lukhanyiso Mthiyane, Josep García Martínez, Sinegugu Khulu, Mbongiseni L. Dlamini, Charlotta Turner, Luke Chimuka

**Affiliations:** 1 Molecular Sciences Institute, School of Chemistry, University of Witwatersrand, 1 Jan Smuts Ave, Braamfontein, Johannesburg, 2000 South Africa; 2 Department of Chemistry, Centre for Analysis and Synthesis, Lund University, 22100 Lund, Sweden; 3 School of Education, University of Witwatersrand, 27 St. Andrews Rd, Parktown, Johannesburg, 2193 South Africa

**Keywords:** Pharmaceuticals, Trimethoprim, Nevirapine, Environmental water monitoring, Environmental health risk

## Abstract

The presence of pharmaceuticals, especially antibiotics, in aquatic environments can significantly contribute to the rise of antibiotic resistance, presenting a critical global public health challenge. This study investigates the presence of four selected antibiotics and an antiretroviral drug (nevirapine) in the Vaal River in South Africa. Grab samples collected at nine sampling sites across four seasons were extracted using solid-phase extraction and analysed using liquid chromatography coupled with triple quadrupole mass spectrometry. The method detection limits ranged from 0.045 ng L^−1^ for trimethoprim to 4.41 ng L^−1^ for ciprofloxacin. A general trend of increasing concentrations was observed from upstream to downstream in all seasons, with autumn and winter showing higher concentrations than spring and summer at most sampling sites. The major contributors to the total concentration across all seasons were trimethoprim (3–76%) and nevirapine (4–48%). The environmental risk assessment showed seasonal variability in the ecological risks posed by the targeted pharmaceuticals, with higher risks observed in autumn and winter. Amongst the studied pharmaceuticals, ciprofloxacin exhibited the highest ecological risk, with its risk quotient evaluated to be 11.67. These results call for action to ascertain the sources and stop their release into the river across the catchment area.

**Supplementary Information:**

The online version contains supplementary material available at 10.1007/s10661-025-14649-4.

## Introduction

Since the discovery of penicillin, the field of pharmaceuticals has grown rapidly, with over 4000 pharmaceuticals now used worldwide for health care in the treatment of humans and animals, as well as promoting the growth of livestock (Branchet et al., 2021). However, the presence of pharmaceuticals in aquatic ecosystems is a growing global concern, as their widespread usage has seen their residues being detected in abundance in aquatic ecosystems (Fonseca et al., 2020). Whilst not traditionally classified as pollutants, these substances are becoming more recognized for their persistence in the environment because of continuous use, resulting in bioaccumulation potential (Wang et al., 2021) and harmful effects on aquatic life and human health (Munzhelele et al., 2024). Unlike conventional pollutants, emerging pollutants (EPs), specifically pharmaceuticals, often resist removal by traditional wastewater treatment methods, leading to continual release into water bodies (Li et al., 2023b). In the event that wastewater is inadequately treated, pharmaceutical residues are discharged into the environment, where they persist. Their persistence in the environment and potential to disrupt ecological processes draw attention to the pressing need to understand their ecotoxicological impacts.

Antifolates, fluoroquinolones (FQs), and antiretrovirals (ARVs) are widely used pharmaceuticals (Du et al., 2023; Kutuzova et al., 2021; Mohan et al., 2021), and their ecotoxicity impacts in aquatic ecosystems pose an emerging threat due to their bioactivity and persistence (Oluwole et al., 2020). FQs (ofloxacin, ciprofloxacin, and norfloxacin) and their metabolites can occur in varying concentrations, which have been seen to be harmful to different organisms: bacteria, fish, and plants (Du et al., 2023; Shen et al., 2023). Over time, these antibiotics can cause cumulative, chronic damage to organisms and environmental health by promoting the development of antibiotic-resistant genes (ARGs) (Sun & Zheng, 2023). Although the data available are limited, ARVs (nevirapine) have also been seen to potentially have various ecotoxicological effects on aquatic organisms, such as mutagenic effects in bacteria or decreased growth rates in fish (Mahaye & Musee, 2022). Similarly, trimethoprim, an antifolate, has been reported to reduce the growth and development of aquatic organisms like microalgae (Mpatani et al., 2021). The ecological risks of these widely used pharmaceuticals emphasise the importance of understanding their impacts, especially in aquatic ecosystems, which have a large percentage of waters receiving pharmaceutical residues due to improper discharge or human excreta (Oluwole et al., 2020).

The common analytical technique for the analysis of pharmaceuticals in aquatic systems is solid phase extraction followed by tandem mass spectrometry (Mosekiemang et al., 2019; Semreen et al., 2019; Sadutto & Picó, 2020). SPE is highly versatile and effective in extracting a wide range of organic compounds, including pharmaceuticals found in complex environmental samples, because of the availability of a wider choice of sorbents (Andrade-Eiroa et al., 2016).

Developing countries like South Africa face challenges with high concentrations of pharmaceutical residues detected in their water systems due to various reasons, such as high urbanisation putting a strain on already poor functioning wastewater treatment facilities, and discharging untreated wastewater into rivers (Munzhelele et al., 2024). The Vaal River, one of South Africa’s largest rivers, supplying water to the country’s economic centre (Gauteng), is under threat as it has recently been reported to be “polluted beyond acceptable limits” (Mnguni, 2022).

Research on water quality in the Vaal River catchment has, to a great extent, explored various contaminants, such as heavy metals, nutrients, and microplastic pollution (Iloms et al., 2020; Ntshalintshali, 2019; Ramaremisa et al., 2022). However, there has been little focus on pharmaceutical contaminants: their presence, seasonal changes, or ecological risks in the Vaal River catchment. Most research on emerging pollutants in South Africa has been carried out in other regions of the country (Ngqwala & Muchesa, 2020), leaving a significant knowledge gap when it comes to pharmaceutical pollution in the Vaal River. There is also a limited understanding of how seasonal changes affect pharmaceutical concentrations and the related ecological risks. This is a critical issue, given that the Vaal River is a key water resource for industry, agriculture, and domestic use in South Africa.

This study aims to address this gap by investigating the presence and seasonal variation of selected pharmaceuticals in the Vaal River catchment. This was done by evaluating their concentrations and assessing the potential ecological risks. As such, this study hopes to provide valuable insights into pharmaceutical contamination and its potential impact on both the environment and human health.

## Materials and methods

### Sampling area

**Fig. 1:**
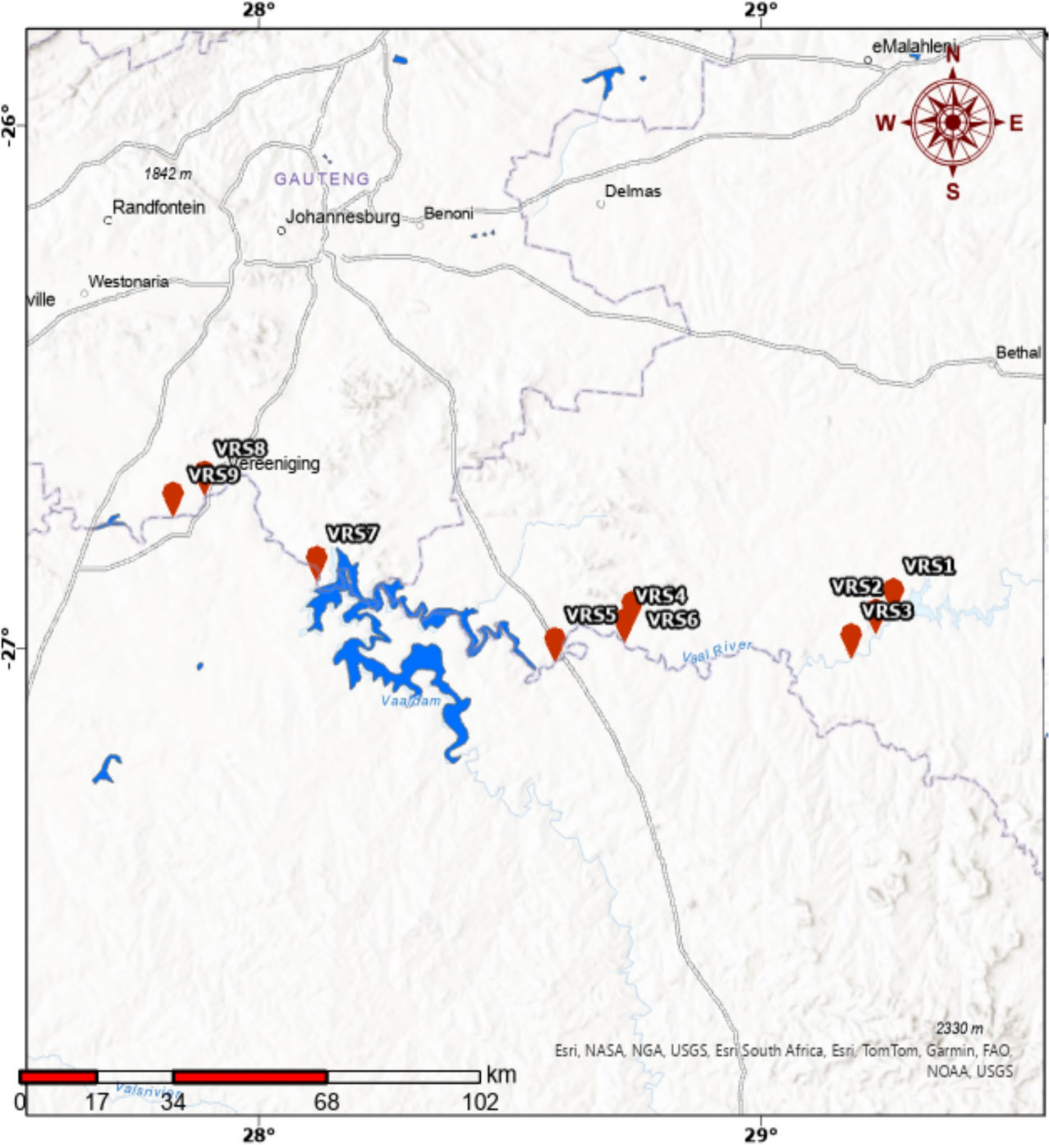
Map showing the locations of the sampling sites used in this study. The river flows through VRS1 upstream through VRS9 downstream

In this study, nine sampling points were identified from point and non-point source pollution along the Vaal River, South Africa, as seen in Fig. 1. These sampling sites were located along the Vaal River Catchment, covering areas in Standerton, Mpumalanga province; Villiers, Free State province; and Vanderbijlpark, Gauteng province. This covered a distance of approximately 309 km of the river. The Vaal River is the third largest in South Africa. It is 1,458 kms long and flows downstream into the Orange River (the biggest River and 2200 km long). The sampling sites were arranged from upstream to downstream, following the westward flow of the river, to assess any changes along the river, especially as it passes through South Africa’s industrial hub. The first sampling point is situated near a drinking water treatment plant (VRS 1, − 26.9357030, 29.2653770), followed by the second sampling point (VRS 2, − 26.9749890, 29.2299370) located next to a wastewater treatment plant and an industrial area. The third sampling point (VRS 3, − 27.0213490, 29.1812500) is situated next to an urban residential area with a township and a tuberculosis (TB) clinic. The first three points are situated in Standerton, covering a distance of about 28 km. The fourth sampling point (VRS 4, − 26.9917870, 28.7298010) is located at the border between the Mpumalanga and Free State Provinces, covering a distance of 71 km from VRS 3. VRS 5 (− 27.0288940, 28.5911220) is located downstream from Villiers (17 km apart from VRS 4), whilst VRS 6 (− 26.9598284, 28.7446919) is located at a tributary of the Vaal River in Villiers, at a distance of 22 km from VRS 5. The seventh sampling point, VRS 7 (− 26.8737580, 28.1189140), is situated at the Vaal Dam outflow in the Gauteng province (83 km from VRS 6), and VRS 8 (− 26.7112010, 27.8920000) and VRS 9 (− 26.7581690, 27.8304000) are in the urban/residential areas in Vanderbijlpark. The last three sampling points are in Vanderbijlpark and cover a distance of about 47 km.

### Sample collection and preparation

Grab samples were collected once in all four seasons during the months of March 2023 (autumn), June 2023 (winter), November 2023 (spring), and February 2024 (summer). The samples were collected into pre-cleaned plastic containers in triplicates from nine sampling sites along the Vaal River Catchment area of South Africa. The samples were then transported to the laboratory in a cooler box, and the physicochemical properties of the water samples were measured immediately upon reaching the laboratory. These properties included pH, conductivity, total dissolved solids (TDS), and salinity. The water samples were then subjected to pre-treatment to remove any particulate matter. Filtration was done using 0.7 µm glass microfiber filters under vacuum to ensure efficient removal of suspended solids. The filtered samples were then stored at 4 °C until further analysis to preserve sample integrity and prevent degradation of analytes. Thereafter, the frozen samples were then shipped to a laboratory at Lund University, Sweden, in a cooler box under ice. Upon arrival, the samples were extracted using an optimised solid-phase extraction method adopted from Madikizela and co-workers (Madikizela et al., 2020). HLB SPE cartridges were first pre-conditioned with 5 mL of methanol, followed by 5 mL of ultrapure water. After measuring the water samples to 200 mL, they were loaded into the SPE cartridges and allowed to flow at a constant flow rate (1–2 drops/sec) by adjusting the vacuum manifold. The cartridges were then washed with 5 mL of a methanol:water solution (10:90% v/v) and dried under vacuum for 1 min. The analytes of interest were eluted using 5 mL of a methanol:water solution (80:20% v/v). The extracts were then evaporated under a gentle stream of nitrogen gas to 1 mL. The concentrated extracts were transferred to 2 mL autosampler vials for HPLC-QqQ-MS analysis. The concentrations in the water samples were obtained using calculations that involved the optimised recoveries from SPE, the final concentration in 1 mL extracts, and the volume of the extracted samples.

### Chemicals and pharmaceutical standards

The analytical standards consisted of trimethoprim (TMP) (≥ 98%), norfloxacin (NOR) (≥ 98%), ciprofloxacin (CIP) (≥ 98%), ofloxacin (OFL) (≥ 99%), and nevirapine (NVP) (≥ 98%) obtained from Merk Life Science (Pty) Ltd (Johannesburg, South Africa). Table S1 shows the target compounds as well as their physicochemical properties. LC–MS-grade (99%) methanol and acetonitrile used to prepare the stock solution were purchased from VWR Chemicals (PA, USA). Formic acid (98%), LC–MS-grade, which was used in the preparation of the eluents A and B for the HPLC–MS/MS analysis, was purchased from Sigma-Aldrich (St. Louis, MO, USA). The nitric acid, which was used during the cleaning process of the sampling bottles and for containers used during physical chemical properties measurements for quality assurance, was also obtained from Sigma-Aldrich (St. Louis, MO, USA). The nitrogen gas used in the evaporation of eluents was purchased from AGA Industrial Gases (Lidingö, Sweden).

### Instrumentation

Ultrapure water, produced through a Milli-Q IQ 7000 Q-Pod from Merk Millipore (MA, USA), was used for diluting stock solutions and preparing working standard solutions. The SPE manifold used for sample preparation of the grab samples was the Biotage VacMaster 10 from Biotage (Uppsala, Sweden). AttractSPE™ HLB SPE cartridges (6 mL/500 mg) were purchased from Anatech Pty Ltd (Johannesburg, South Africa). Whatman® filters and borosilicate glass grade GF/F filter discs with a 0.7 μm pore size purchased from Merk Life Science (Pty) Ltd (Johannesburg, South Africa) were used for filtering water samples.

Water quality parameters were measured using a Multiparameter Bluetooth® Portable pH/EC/OPDO Meter (HI98494) supplied by Hanna Instruments (Johannesburg, South Africa).

For the separation and analysis of the target compounds, an HPLC system (Agilent 1290 Infinity II LC) from Agilent Technologies (Santa Clara, CA, USA) was used. The system consisted of a 1290 Infinity II High-Speed binary pump and a 1290 Infinity II multisampler. The UHPLC was coupled to a 6495B triple quadrupole mass spectrometer system from Agilent Technologies (Santa Clara, CA, USA). Chromatographic separation of the analytes was achieved on a Luna C_18_ (2)-HST column (100 mm × 2 mm, 2.5 μm) from Phenomenex (Torrance, CA, USA). For the collection and processing of qualitative data, Agilent MassHunter Qualitative Navigator (B.08.00) was used.

### HPLC-QqQ-MS method

The analysis was performed using 0.1% formic acid in deionised water as solvent A and 0.1% formic acid in a methanol and acetonitrile (80:20) solution as solvent B in gradient elution mode. The gradient started at 10% of solvent B at 0.00 min, which gradually increased to 15% at 2.00 min, further increased to 30% at 6.00 min, and then increased to 90% at 10.00 min. It was kept constant at 90% until 12.00 min and then decreased back to 10% at 14.50 min, where it was kept constant until 16.00 min. A flow rate of 0.4 mL min^−1^ and an injection volume of 5 μL were used throughout the analyses.

Tandem-MS analysis was carried out with an ESI interface. The analyses were done in positive-ion (PI) mode for all the analytes investigated. Following the selection of the precursor ions, product ions were obtained at a series of collision energies and selected according to the fragmentations that produced a useful abundance of fragment ions. The collision energies and transitions chosen for the multiple reaction monitoring (MRM) experiment are listed in Table 1. For all the targeted analytes, the optimal collision energy was determined by multiple experiments, which were done by injecting the standards into the instrument at various collision energies and choosing the collision energies that gave the most informative fragmentation patterns. The fragmentor and cell accelerator voltages were kept constant at 380 V and 4 V, respectively, for all analytes. Each analyte of interest was analysed by MRM, using the two highest characteristic precursor ion/product ion transitions (Table 1) for quantifying (in bold) and identifying the compounds, respectively.

**Table 1:** LC–MS/MS conditions, in MRM mode, for analysis of target analytes

Pharmaceutical compound	Parent ion (m/z)	Product ion (m/z)	Collision energy (eV)
Trimethoprim	291	**230** 261	25 25
Ofloxacin	362	**318** 261	15 25
Norfloxacin	320	**302** 276	15 15
Ciprofloxacin	332	**314** 231	20 35
Nevirapine	267	**80** 53	50 62

### Quality assurance

Quality assurance procedures were implemented to ensure the reliability of results.

### Cleaning procedures

All the containers used in this study were thoroughly washed and prepared as follows: the containers were first rinsed with tap water to remove any visible residues, then scrubbed gently using a soft brush and mild detergent to ensure they were clean. After scrubbing, they were rinsed again to remove all detergent residues. To eliminate any trace contaminants, the containers were soaked in 10% nitric acid for 24 h and then rinsed thoroughly with ultrapure water. Next, they were rinsed with methanol to remove any organic contaminants and left to air dry. Finally, the containers were given one more rinse with ultrapure water and allowed to dry completely in a clean environment.

To prevent any cross-contamination, the solid phase extraction (SPE) manifold was cleaned prior to use. The cleaning procedure for the SPE manifold components mirrored the thorough cleaning procedures outlined for the sample glass containers, ensuring the removal of any potential contaminants.

### Method validation

Method validation parameters, such as recovery, trueness, precision, and sensitivity, as well as matrix effects, were evaluated. A stock solution with a mixture of the selected pharmaceuticals was prepared to 1000 μg mL^−1^ in pure methanol and acetonitrile (50:50% v/v), which took into account the solubility of the individual pharmaceutical compound. The prepared stock solution was stored at 4 °C and later used for working standard solutions by dilution using ultrapure water at a range of concentrations.

To evaluate the external calibration curves, the coefficient of determination (*R*^2^) was determined as an indicator of linearity in the concentration ranges of 1–50.0 μg L^−1^.

The instrumental limit of detection (LOD) and limit of quantification (LOQ) were measured by injection of a standard solution containing all target compounds at 1 μg L^−1^. The signal-to-noise (S/N) ratios were obtained directly from the instrument’s chromatographic data, where the noise level was calculated from a baseline region free of analytical peaks. The LOD and LOQ were expressed as the concentration relating to a S/N of 3 and 10, respectively. These values were calculated using a standard S/N-based approach, where the observed S/N ratio of the standard was used to estimate the concentration corresponding to the target S/N thresholds. Calculations were done individually for each compound based on the extracted ion chromatograms from the mass spectrometer.

The matrix-matched method detection limits (MDL) and method quantitation limits (MQL) were derived from the instrumental LOD and LOQ, taking into account the recovery from SPE and the resulting enrichment factor. The instrumental LOD and LOQ values were divided by the enrichment factors to obtain the MDL and MQL, respectively (Megersa et al., 2001).

For the recovery calculations from SPE, procedural and sample blanks were used to evaluate the SPE method's performance by extracting blank samples first, followed by spiking the samples at two different concentrations (5 and 10 μg L^−1^) in triplicates. The recovery percentages and %RSD were used to evaluate the trueness and precision of the method.

Matrix effects were determined according to **Eq. **1**,** which was adapted from an equation by Nasiri et al. (2021):

1 $$\text{Matrix effect }\left(\text{\%}\right)=\left(\frac{\text{\% recovery in river water}}{\text{\% recovery in ultrapure water}}-1\right)\times 100$$

### Environmental health risk assessment

The environmental health risk assessment was carried out using a ratio of the maximum environmental concentration (MEC) to the predicted no-effect concentration (PNEC) (Ncube et al., 2021). This approach considered PNEC-resistance, which is related to the development of antibiotic resistance in aquatic organisms. PNEC-resistance values were based on the lowest minimal inhibitory concentrations (MICs) that could trigger antibiotic resistance in aquatic organisms and were obtained from a study by Bengtsson-Palme and Larsson (2016).

Although compounds such as nevirapine can contribute to the selection of antibiotic resistance, it was not included in this environmental health risk assessment. This is because nevirapine, being a non-antibiotic, does not have a PNEC-resistance value, which is typically used to assess the potential for antibiotic resistance development.

To estimate the risk quotient (RQ), the PNEC values were compared to the measured environmental concentration (MEC) of the target compounds. The RQ was calculated using **Eq. **2 (Lin et al., 2023; Ncube et al., 2021):

2 $$RQ=\frac{MEC}{PNEC}$$

where MEC is the maximum environmental concentration, and PNEC is the predicted no-effect concentration. The risk levels were categorised as follows: RQ ≤ 0.1 indicates low environmental risk, an RQ between 0.1 and 1 suggests moderate risk, and an RQ > 1 signifies high environmental health risk (Ren et al., 2023).

### Statistical analysis

Statistical analysis was conducted to determine the distribution and seasonal variation of the selected emerging pollutants, with a focus on identifying significant differences across sampling sites and seasons. A two-factor analysis of variance (ANOVA) without replication was used to assess variations in total analyte concentrations across seasons per sampling site, with the *p*-values used to assess significance. A *p* < 0.05 indicates meaningful differences, whilst a *p* > 0.05 suggests variability that is not statistically significant. Pearson correlation tests were performed to explore the relationships between the concentrations of the target analytes and water parameters of all seasons.

These analyses were carried out using the XLSTAT statistical software (ADDINSOFT, NY, USA) and the OriginLab software.

## Results and discussions

### Analysis of water quality indicators

**Fig. 2:**
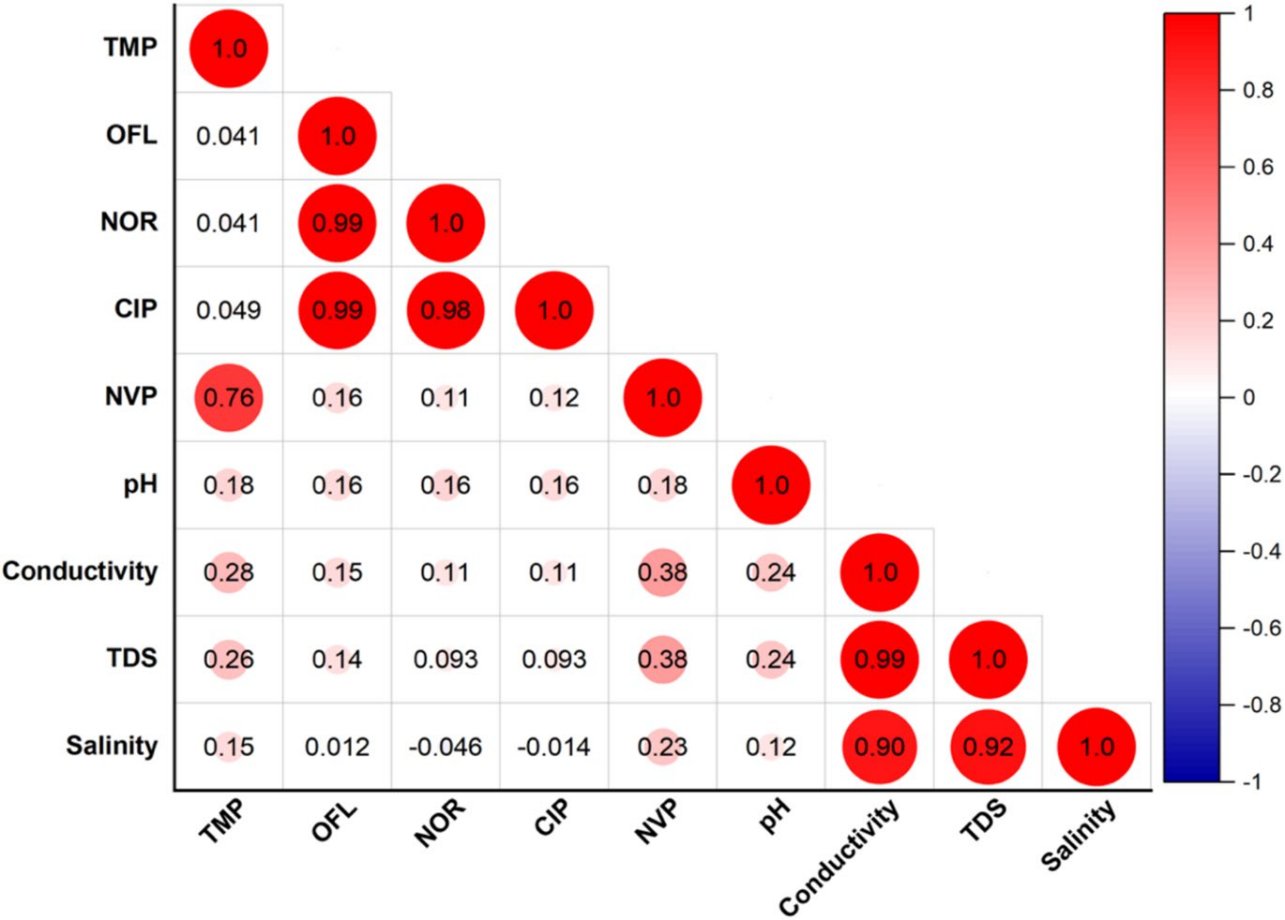
Pearson correlation plot of target compounds and water quality parameters for all seasons

The physicochemical analysis revealed notable seasonal and spatial variability in the water quality parameters across the sampling sites. The measured physicochemical properties of the river water (Table S2) were compared to the South African National Standards (SANS) for drinking water, as many South Africans living in rural areas make use of untreated river water for drinking and domestic use due to inadequate water supply (Edokpayi et al., 2017). The pH values across all seasons were within the SANS limits, which indicates no immediate concerns due to acidity or alkalinity. However, conductivity exceeded the limits in all the seasons, suggesting anthropogenic influences. Total dissolved solids (TDS) levels remained within the SANS limits throughout all seasons, ranging from 75.7 to 462.0 mg L^−1^, which indicated moderate ion concentrations. Electrical conductivity and total dissolved solids exhibited a consistent increase from upstream to downstream across all four seasons. In winter and spring, the electrical conductivity was above the SANS limits across all sampling sites, whereas in autumn and summer, the electrical conductivity exceeded the SANS limits from sampling site VRS 2 until VRS 9. Higher conductivity was observed in spring and summer, ranging from 148.4 to 845.3 μS/cm. Similarly, total dissolved solids ranged from 92.0 to 462.0 mgL^−1^ in both spring and summer. These seasons are known to be wet seasons due to increased rainfall. Therefore, this phenomenon may be attributed to seasonal runoff from surrounding areas. This may then lead to increased concentrations of dissolved ions in the river, which influences conductivity and TDS (Thirumalini & Joseph, 2009). The strong correlation between conductivity and total dissolved solids, confirmed by the Pearson correlation matrix (Fig. 2), further validates this relationship. The Pearson correlation coefficient (r = 0.99) highlights the significant influence of dissolved ion concentrations on these parameters. Salinity remained low during all seasons with little variation, as salinity was ≤ 0.46 g L^−1^. Low salinity is typically expected in freshwater systems unless there are influences like industrial discharges or agricultural runoff, which may elevate salt levels locally (Jiang et al., 2022). During dry seasons (autumn and winter), decreased water levels as a result of reduced rainfall and water flow may be observed, which may explain the trends seen in pH values. In this sense, the results from pH measurements exhibited a general neutral to slightly basic condition. Although it is a slight variation, an increase in pH during autumn and winter could be indicative of diminished inputs of acidic substances, such as agricultural runoff or industrial discharges from the surrounding environment, leading to more alkaline conditions observed (Jiang et al., 2022).

### Method validation

The obtained method validation parameters are given in Table 2**.** The correlation coefficients (*R*^2^) obtained from calibration curves ranged from 0.9909 to 0.9994, indicating good overall linearity of the target compounds. To determine the trueness of the analytical method, recovery experiments were conducted for each of the target compounds. The recoveries of both the procedural and sample blanks were performed by spiking at 5 and 10 ng mL^−1^. Acceptable recoveries, ranging from 70.2% to 105.5%, were obtained for all the target pharmaceuticals. RSD values were also obtained after the spiked samples were run in triplicate. The RSD values were ≤ 3.33%, which indicated good repeatability and reliability. This analytical method showed detectability to all the target compounds as the MDLs were estimated to range from 0.051 ng L^−1^ for trimethoprim to 4.50 ng L^−1^ for ciprofloxacin, whilst the MQLs ranged from 0.17 ng L^−1^ for trimethoprim to 14.98 ng L^−1^ for ciprofloxacin. In a study reported by Čizmić et al. (2017) the MDL values for trimethoprim and ciprofloxacin were reported as 1.90 ng L^−1^ and 30.60 ng L^−1^, respectively, whilst the MQL values were 6.30 ng L^−1^ and 102.00 ng L^−1^, respectively. The lower MDL and MQL values obtained in this study indicate enhanced detectability towards these two target compounds compared to the study by Čizmić et al. (2017). Similarly, Maia et al. (2020) reported MDL values of 6.70 ng L^−1^ for ofloxacin and 36.30 ng L^−1^ for norfloxacin, with corresponding MQL values of 22.30 ng L^−1^ and 121.00 ng L^−1^, respectively. These values were higher than those reported in this study, where the MDL values were 0.37 ng L^−1^ and 2.54 ng L^−1^ for ofloxacin and norfloxacin, and the MQL values were 1.24 ng L^−1^ and 8.47 ng L^−1^ for ofloxacin and norfloxacin, respectively. This indicated higher detectability for ofloxacin and norfloxacin as well.

**Table 2:** Method validation parameters

Pharmaceutical compounds	*R*^2^	MDL (ng L^−1^)	MQL (ng L^−1^)	% Recovery ± %RSD				% ME
				**Spiked ultrapure water (μg L**^**−1**^**)**		**Spiked river water (μg L**^**−1**^**)**		
				**5**	**10**	**5**	**10**	
**Trimethoprim**	0.9994	0.051	0.17	98.74 ± 1.46	95.07 ± 2.24	82.53 ± 1.08	92.51 ± 1.00	− 10
**Ofloxacin**	0.9909	0.37	1.24	75.23 ± 1.33	90.38 ± 1.10	103.66 ± 2.49	105.45 ± 0.94	26
**Norfloxacin**	0.9966	2.54	8.47	79.71 ± 0.48	74.66 ± 1.53	102.63 ± 0.89	103.04 ± 0.15	33
**Ciprofloxacin**	0.9971	4.50	14.98	91.52 ± 0.57	93.41 ± 0.44	98.35 ± 1.12	97.94 ± 3.02	6
**Nevirapine**	0.9992	1.63	5.43	95.73 ± 3.33	70.23 ± 2.51	96.52 ± 2.13	78.72 ± 1.32	6

Matrix effects were also determined to measure whether the environmental matrix affected the accuracy of the analytical method for the target compounds in river water. Generally, matrix effects occur when components present in the sample, such as organic matter or non-target compounds, interfere with the ESI ionisation process during HPLC–MS/MS analysis or solid phase extraction, thus resulting in signal suppression or enhancement of the target compounds (Rivera-Jaimes et al., 2018). For matrix effect correction, the preferred method is through employing an internal standard. However, in the absence of an internal standard like in this work, an external sample calibration may be suitable to compensate matrix effects from moderately loaded samples such as surface waters, groundwaters and wastewater treatment plant effluents (Stüber & Reemtsma, 2004).

The matrix effects observed in this study ranged from − 10 to 33%. One of the five target compounds, trimethoprim, showed ion suppression of 10%. Norfloxacin was the most affected by the matrix effects, as it showed an ion enhancement of 33% (Table 2). The matrix effects exhibited for ofloxacin and ciprofloxacin were slight to moderate signal enhancement, with values of 26% and 6%, respectively. The matrix effect for nevirapine in this study was minimal, with a signal enhancement of 6%. In contrast, Ngumba et al. (2016) reported significantly higher signal enhancement for nevirapine at 90.44%. Whilst both studies showed the presence of matrix effects (signal enhancement), the difference in the magnitude can be due to differences in sample composition and environmental conditions.

### Occurrence of pharmaceuticals using solid phase extraction

**Fig. 3:**
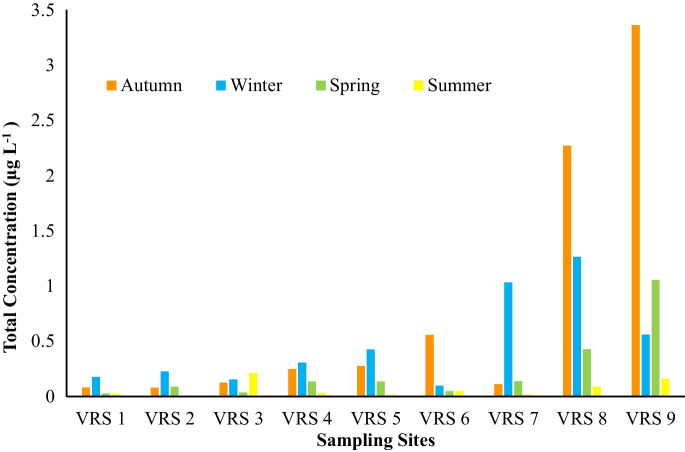
Total concentration of analytes detected across various sampling sites in all 4 seasons

Figure 3 shows the total concentrations of the targeted analytes detected at the nine sampling sites across four seasons. The detection of emerging pollutants in surface water can vary significantly across seasons due to a range of environmental and man-made factors (Archana et al., 2017). Understanding these seasonal fluctuations is important for assessing the persistence and behaviour of these compounds in the environment. The general trend that was observed was an increase in the total concentration of the analytes going downstream, reaching 3.36 µg L^−1^ in Autumn (VRS 9).

The seasons that showed higher concentrations at all sampling sites were autumn and winter, when compared to spring and summer. This could be because autumn and winter are dry seasons, and there is little to no rain experienced, which could concentrate pollutants in the river as there is limited water inflow, resulting in higher concentrations (Mosekiemang et al., 2019).

A two-factor without replication analysis of variance (ANOVA) was performed to assess the statistical variance of the total concentrations of the target analytes per sampling site across four seasons. The results indicate a *p* > 0.05, suggesting that there are no statistically significant differences in the total concentrations of the target compounds within each sampling site across the different seasons. The absence of any statistically significant differences could mean that the concentrations of the targeted compounds are statistically relatively consistent across seasons for each sampling site. This could imply that the sources of these pollutants are widespread or uniformly distributed across the area and are not strongly influenced by seasonal variation or specific locations within the study area. For example, whilst there is a notable difference in the total concentration between VRS 1 and VRS 9, this difference was not assessed by ANOVA, which only compared the total concentrations within the same sampling site across different seasons. Although there was no statistically significant difference seen using ANOVA, minor variations in the total concentrations are observed in Fig. 3**.**

**Fig. 4:**
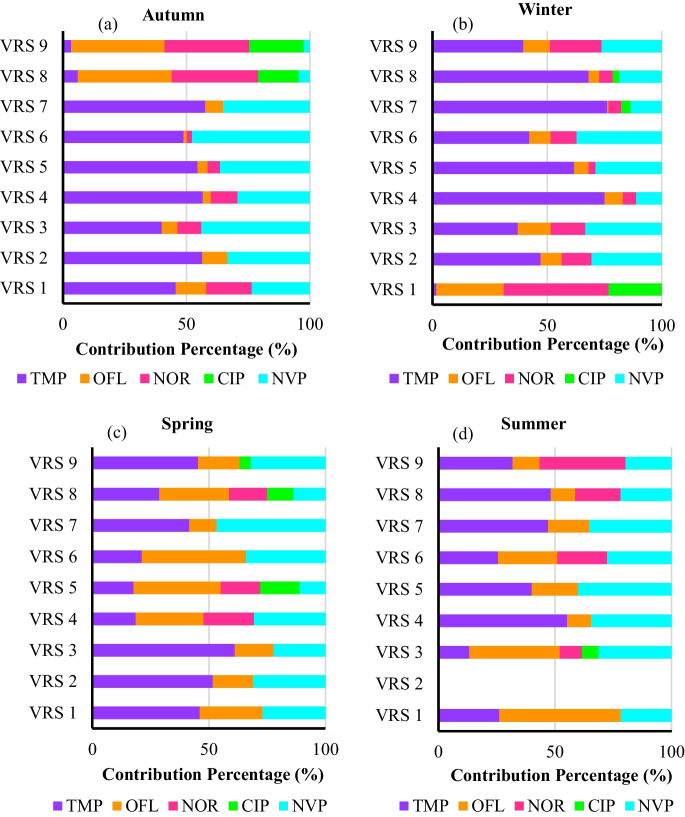
Contribution percentage (%) of the targeted compounds to the total concentration across various sampling sites in all seasons: **a** autumn, **b** winter, **c** spring, and **d** summer

The contribution percentages of the targeted compounds across the different sampling points reveal potential sources of pollution. These patterns likely reflect specific activities, land use, or environmental factors in each area, as indicated by the compounds in relatively higher concentrations at certain sites. This suggests that localised pollution sources, such as agriculture and wastewater discharge, may influence the distribution of these pollutants. As seen in Fig. 4a and b, in both seasons, autumn and winter, a similar trend was observed; trimethoprim and nevirapine were the main contributors to the total concentrations in most of the sampling sites, with exceptions to VRS 8 and 9 in autumn and VRS 1 in winter.

A similar trend was observed in spring and summer (Fig. 4c and d**)**, where trimethoprim and nevirapine were the main contributors to the total concentrations at all the sampling sites. These two compounds are known for their environmental stability, which allows them to persist in aquatic systems for extended periods (Adeola & Forbes, 2022). Their chemical structure makes them resistant to natural degradation processes, such as biodegradation and photodegradation, explaining their consistent presence across sampling sites (Zhang et al., 2023). This relationship was further confirmed by the Pearson correlation matrix (Fig. 2), which indicated a strong positive correlation between trimethoprim and nevirapine (*r* = 0.76). This correlation might suggest that the two compounds may share similar sources. VRS 2 in the summer season was not sampled as there was no way to access the sampling site due to construction, which was occurring at the time of sampling. Therefore, there is no data available for this sampling site in the summer.

Interestingly, ofloxacin and norfloxacin showed a noticeable increase in their contribution percentage during spring and summer compared to autumn and winter in the majority of the sampling sites. This could be due to several factors. Warmer months often coincide with higher incidences of bacterial and viral infections, leading to greater use of these antibiotics. Increased usage means more of these pharmaceuticals end up in wastewater. Additionally, untreated or partially treated wastewater entering the river during these seasons, as observed during sampling, plays a significant role in introducing these compounds into the water. The Pearson correlation matrix (Fig. 2) supported this observation, showing a remarkably strong correlation between ofloxacin and norfloxacin, with a correlation coefficient of *r* = 0.99. This near-perfect correlation indicates that these two compounds are likely introduced into the river system through similar pathways or sources.

Environmental conditions in spring and summer may also influence how these pharmaceuticals behave in the river. A study by Bhlokhina et al. (2016) revealed that increasing temperature increases the solubility of fluoroquinolones (including norfloxacin and ciprofloxacin). As such, warmer temperatures (in summer and spring) can enhance their solubility and mobility, making it easier for them to disperse (Bhlokhina et al., 2016). At the same time, higher microbial activity during these months might lead to some breakdown of these compounds, potentially forming transformation products. Whilst by-products were not analysed in this study, they could be an important factor to explore in the future.

In this work, it is noted that across all seasons the highest obtained total contribution of all target pharmaceuticals is 3.36 µg L^−1^ (Fig. 3), with trimethoprim and nevirapine being the highest contributors across all seasons (Fig. 4). These results are comparable to those obtained in the literature (Table 3), where the data show higher concentrations of these two compounds detected across the globe as opposed to the other three target compounds. In the work conducted in Kenya by Wanjeri et al. (2023), trimethoprim recorded high concentrations of up to 8.49 and 39 µg L^−1^ during the wet and dry seasons, respectively. Similarly, nevirapine shows prevalence compared to other compounds, with up to 1.80 and 10.18 µg L^−1^ in the wet and dry seasons (Wanjeri et al., 2023). Although their values are extremely high compared to the values in this study, due to the abuse of antibiotics in Kenya (Wanjeri et al., 2023), their data correlates with the results from this work, where higher contributions of nevirapine and trimethoprim are observed during the autumn and winter seasons (dry season). In addition, a study conducted in China found concentrations of ofloxacin and norfloxacin to be 0.192 µg L^−1^ and 0.131 µg L^−1^, respectively (Chen et al., 2018). This literature data ranks these two compounds as the second highest contributors amongst the selected target compounds (Table 3), which also correlates with the findings of this work.

**Table 3:** Concentrations of selected target pharmaceuticals obtained from other parts of the world and in South Africa

Pharmaceutical compounds	Country	Methods	Concentration (µg L^−1^)	References
Trimethoprim	Portugal	SPE HPLC–MS	< 0.040	(Gorito et al., 2025)
	China	SPE UPLC-MS	0.0954	(Chen et al., 2018)
	Kenya	SPE HPLC–MS	0.90–39	(Wanjeri et al., 2023)
Ofloxacin	Portugal	SPE HPLC–MS	< 0.040	(Gorito et al., 2025)
	China	SPE UPLC–MS	0.002	(Hu et al., 2024)
	China	SPE-UPLC-MS	0.192	(Chen et al., 2018)
Norfloxacin	China	SPE UPLC–MS	0.002	(Hu et al., 2024)
	China	SPE UPLC-MS	0.131	(Chen et al., 2018)
Ciprofloxacin	China	SPE UPLC-MS	0.064	(Chen et al., 2018)
	Spain	SPE HPLC–MS	0.001	(Mejías et al., 2023)
Nevirapine	South Africa	SPE HPLC–MS	0.007–0.166	(Nibamureke & Barnhoorn, 2025)
	Kenya	SPE HPLC–MS	0.270–10.18	(Wanjeri et al., 2023)
	South Africa	MASE-MIP HPLC–MS	0.001–0.002	(Khulu et al., 2022)

### Environmental health risk assessment

**Fig. 5:**
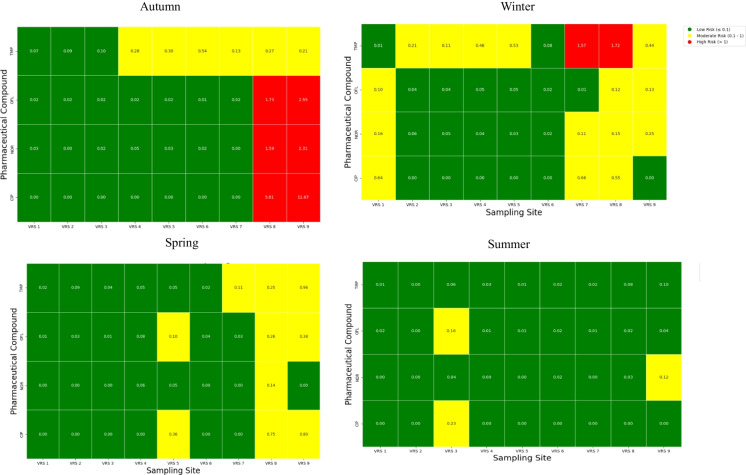
Heatmap of risk quotients for the target compounds across seasons: autumn, winter, spring, and summer

The environmental health risk assessment data is presented in Fig. 5. The estimations of the RQ values revealed variations for the target compounds, seasonally and spatially. In Autumn, trimethoprim showed low to moderate risk across the sampling sites, whilst fluoroquinolones (ofloxacin, norfloxacin, and ciprofloxacin) showed low to high risk across the sampling sites. A high risk for the fluoroquinolones was observed downstream, which highlights their potential to cause harmful ecological effects, with ciprofloxacin exhibiting the highest RQ value of 11.67. This aligns with findings by Li et al., (2023a) who reported elevated RQ values for ciprofloxacin, which were higher than 12.2.

Seasonal variations in the risk levels observed in this study align with the findings of Lin et al. (2023), who reported higher RQ values of pharmaceuticals, such as norfloxacin and ofloxacin, in autumn than in summer. In contrast, spring and summer exhibited lower RQ values, consistent with increased rainfall and dilution in this study. However, the highest moderate risk associated with ciprofloxacin in summer (RQ = 0.23 at VRS 3) highlights the persistence of ciprofloxacin (Mahmood et al., 2019), even with increased water flow conditions.

Trimethoprim showed a different seasonal risk profile, with RQ values of 1.57 and 1.72 reported in winter. This higher risk in winter might be due to specific factors occurring at the sites, such as the discharge of untreated wastewater or increased pharmaceutical use during this period.

## Conclusion

This study highlights the occurrence, seasonal variation, environmental health risk, and persistence of emerging pollutants, particularly pharmaceuticals, in the Vaal River Catchment area. Modern analytical techniques, such as solid-phase extraction and HPLC-QqQ-MS, enabled the detection of the targeted compounds at trace levels. These findings confirm persistent contamination of surface waters, with trimethoprim and nevirapine having the highest percentage contribution to the contamination in all seasons. This highlights the necessity of improved wastewater management practices to reduce contamination. The results of the environmental risk assessment revealed evidence of the environmental risk posed by emerging pollutants, particularly pharmaceuticals, in aquatic environments. This risk is heightened during periods of low water flow, emphasizing the impact of seasonal variability on pollutant concentrations and distribution.

The study recommends continued monitoring to assess the environmental impact of these pollutants over time, advanced risk assessments considering the cumulative effects of multiple contaminants for effective ecological management, tracing the actual pollution sources, and targeted strategies to reduce the pollution.

## Supplementary Information

Below is the link to the electronic supplementary material.

ESM1 (DOCX. 204 KB)

## Data Availability

No datasets were generated or analysed during the current study.
