# Supplementary material for: Seasonal analysis and environmental risk assessment of selected emerging pollutants in the Vaal River catchment area of South Africa
Source: Environ Monit Assess. 2025 Oct 29;197(11):1264. doi: 10.1007/s10661-025-14649-4 (PMC12572104; doi:10.1007/s10661-025-14649-4)
Supplement: Supplementary file 1 — (DOCX. 204 KB) [file 10661_2025_14649_MOESM1_ESM.docx]

**Supplementary Data**

| Pharmaceutical compounds | Empirical formula | Molecular structure | Molecular weight  (g mol^-1^) | Solubility in water  (mg L^-1^) | References | pKa | References |
| --- | --- | --- | --- | --- | --- | --- | --- |
| Trimethoprim | C_14_H_18_N_4_O_3_ | 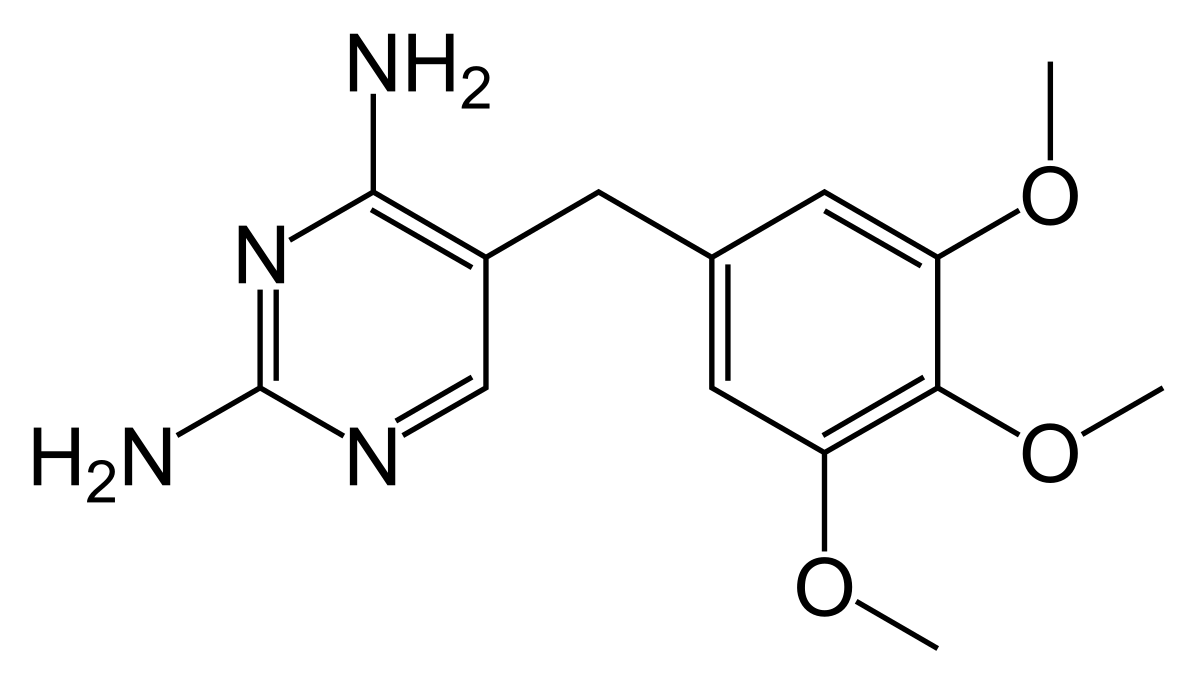 | 290.32 | 400 | Yalkowsky & Dannenfelser, 1992 | 3.23; 6.76 | Perrin, 1965 |
| Norfloxacin | C_16_H_18_FN_3_O_3_ | 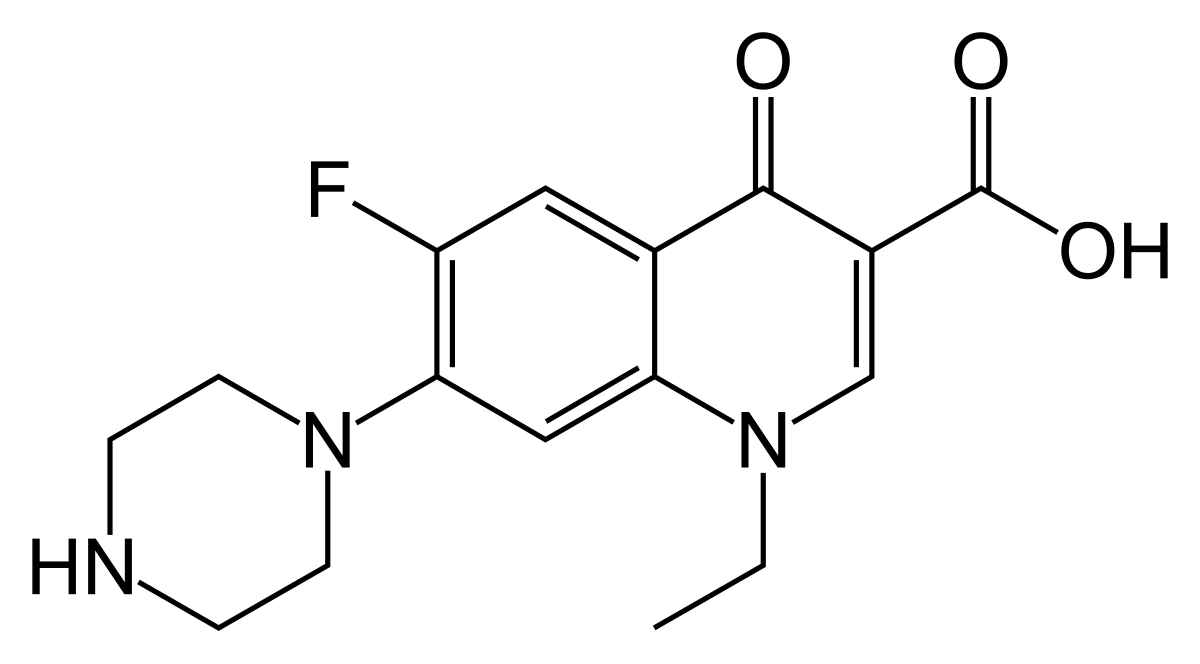 | 319.33 | 280 | O'Neil, 2006 | 6.34; 8.38 | O'Neil et. al, 2006 |
| Ciprofloxacin | C_17_H_18_FN_3_O_3_ | 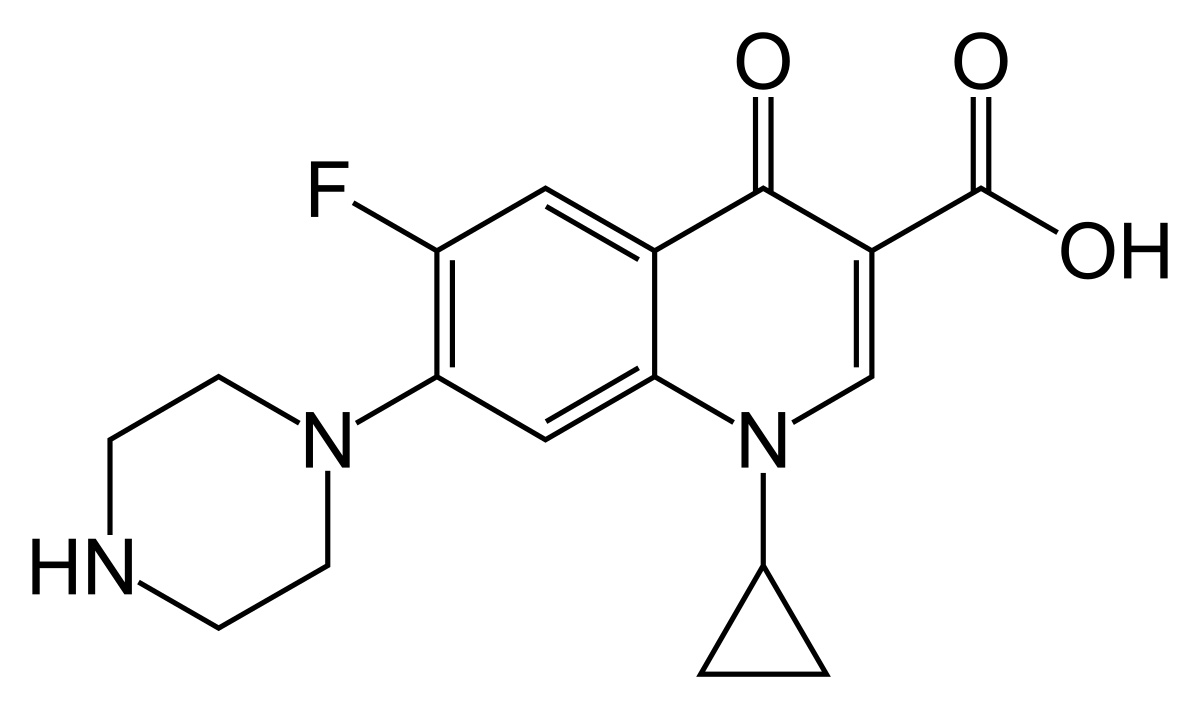 | 331.35 | 30000 | Nowara et al, 1997 | 6.09; 8.74 | Torniainen et al, 1996 |
| Nevirapine | C_15_H_14_N_4_O | 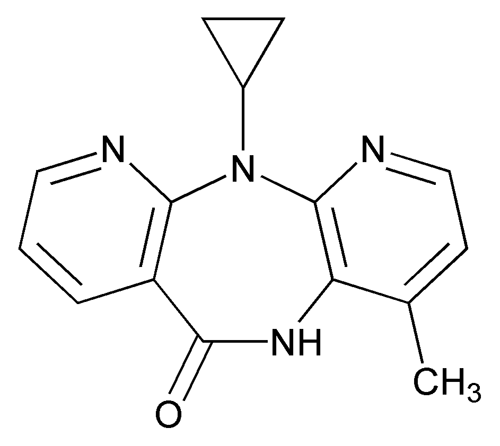 | 266.30 | 100 | Nehal et al., 2003 | 2.8 | Sarkar et al., 2008 |
| Ofloxacin | C_18_H_20_FN_3_O_4_ | 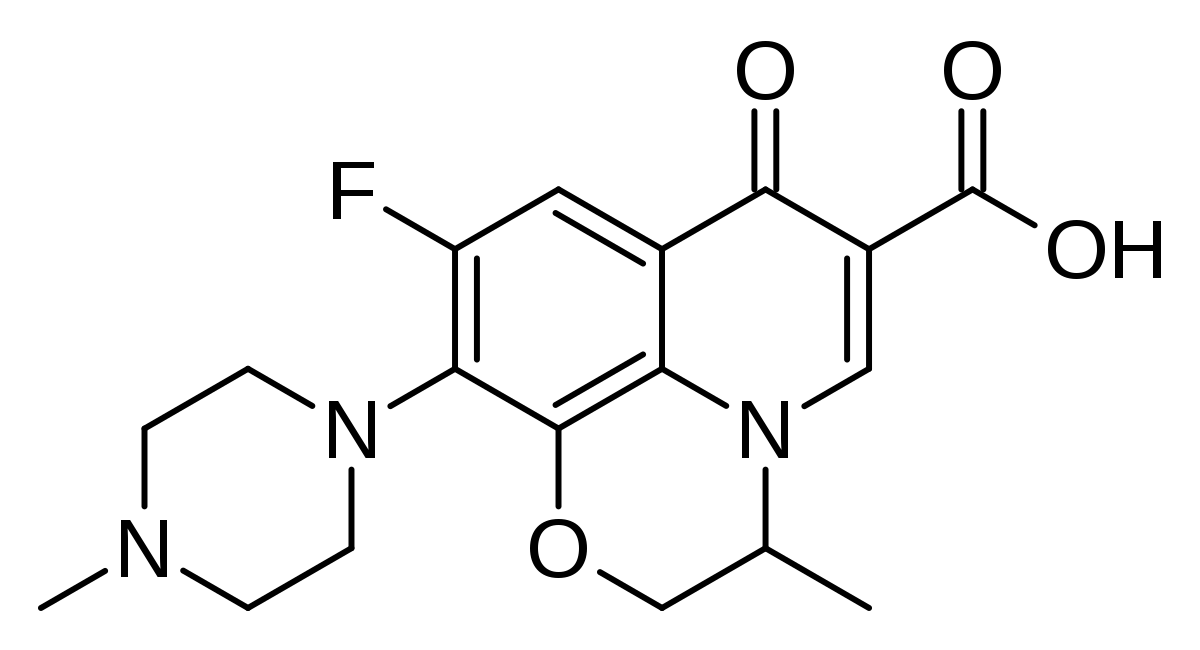 | 361.37 | 28300 | Lewis et al., 2016 | 5.97; 9.28 | Tolls, 2001 |

**Table S1.** Physicochemical properties of targeted analytical compounds

**Details of the sampling sites**

- The first sampling point (VRS 1, Co-ordinates: -26.9357030, 29.2653770 ) was located at the start of the town in Standerton, after the Grootdaai Dam, near a drinking water treatment plant that supplies water to the whole town.
- The second sampling point (VRS 2) was located near a wastewater treatment plant and other factories and industrial plants.
- The third sampling point (VRS 3) was located where the river finally exits Standerton town, at the townships and suburbs, near a tuberculosis (TB) clinic that may discharge its effluent into the river.
- The fourth sampling point (VRS 4) was found at the Free State-Mpumalanga border, upstream of the town of Villiers.
- The fifth sampling point (VRS 5) was located downstream of Villiers town.
- The sixth sampling point (VRS 6) was located at a small river before it joined the Vaal River. The understanding is that this tributary, called the Watervalrivier, may impact the water quality of the main river.
- The seventh sampling point (VRS 7) was located at the outflow of the Vaal Dam as the Vaal River exits the dam.
- The eighth sampling point (VRS 8) was found opposite the middle of Vanderbijlpark town, with townships and suburbs nearby.
- The ninth and final sampling point (VRS 9) was found opposite the end of Vanderbijlpark town near a recreational park.

**Table S2.** Physicochemical properties of water samples across all seasons.

|  | **pH** | **Conductivity**  **(μS/cm)** | **TDS**  **(** **mg L^-1^)** | **Salinity**  **(** **g L^-1^)** |
| --- | --- | --- | --- | --- |
| **SANS** | ≥ 5 - ≤ 9.7 | ≤170 | ≤1200 | - |
| **Autumn** | 7.55 - 8.79 | 151.19 – 466.85 | 75.66 – 233.35 | ND – 0.17 |
| **Winter** | 8.18 - 8.79 | 176.62 – 794.44 | 95.20 – 397.09 | ND – 0.33 |
| **Spring** | 7.40 – 8.52 | 174.41 – 845.28 | 91.98 – 462.04 | 0.08 – 0.46 |
| **Summer** | 7.41 – 8.40 | 148.38 – 828.58 | 106.72 – 414.48 | 0.01 – 0.38 |

**References**

Lewis, K. A., Tzilivakis, J., Warner, D., & Green, A. (2016). An international database for pesticide risk assessments and management. *Human and Ecological Risk Assessment: An International Journal, 22*(4), 1050–1064. https://doi.org/10.1080/10807039.2015.1133242

Sarkar, M., Perumal, O. P., & Panchagnula, R. (2008). Solidstate characterization of nevirapine. *Indian Journal of Pharmaceutical Sciences, 70*(5), 619–630. https://doi.org/10.4103/0250-474X.45401

O’Neil, M. J., Heckelman, P. E., Koch, C. B., & Roman, K. J. (Eds.). (2006). The Merck Index: An encyclopedia of chemicals, drugs,

and biologicals (14th ed.). Hoboken, NJ: John Wiley & Sons, Inc. ISBN13 978-0-911910-001

Nowara, A., Burhenne, J., & Spiteller, M. (1997). Binding of fluoroquinolone carboxylic acid derivatives to clay minerals. *Journal of Agricultural and Food Chemistry, 45*(4), 1459–1463. https://doi.org/10.1021/ jf960 6138

Perrin, D. D. (1965). *Dissociation constants of organic bases in aqueous solution*. Butterworth.

Tolls, J. (2001). Sorption of veterinary pharmaceuticals in soils: A review. *Environmental Science & Technology, 35*(17), 3397–3406. https:// doi.org/10.1021/es001471r

Yalkowsky, S. H., & Dannenfelser, R. M. (1992). *The AQUASOL database of aqueous solubility* (Ver. 5). University of Arizona, College of Pharmacy.

Torniainen, K., Tammilehto, S., & Ulvi, V. (1996). The effect of pH, buffer type and drug concentration on the photo degradation of ciprofloxacin. *International Journal of Pharmaceutics, 132*(1–2), 53–61. https://doi.org/10.1016/0378- 5173(96) 04414-2
